# Inclusion Complexes of Rifampicin with Native and Derivatized Cyclodextrins: *In Silico* Modeling, Formulation, and Characterization

**DOI:** 10.3390/ph15010020

**Published:** 2021-12-24

**Authors:** Qonita Kurnia Anjani, Juan Domínguez-Robles, Emilia Utomo, María Font, María Cristina Martínez-Ohárriz, Andi Dian Permana, Álvaro Cárcamo-Martínez, Eneko Larrañeta, Ryan F. Donnelly

**Affiliations:** 1 Medical Biology Centre, School of Pharmacy, Queen’s University Belfast, 97 Lisburn Road, Belfast BT9 7BL, UK; qanjani01@qub.ac.uk (Q.K.A.); j.dominguezrobles@qub.ac.uk (J.D.-R.); eutomo01@qub.ac.uk (E.U.); acmartinez@live.cl (Á.C.-M.); 2 Department of Pharmaceutics, Faculty of Pharmacy, Hasanuddin University, Makassar 90245, Indonesia; andi.dian.permana@farmasi.unhas.ac.id; 3 Department of Pharmaceutical Technology and Chemistry, Faculty of Pharmacy and Nutrition, University of Navarra, Irunlarrea s/n, 31080 Pamplona, Spain; mfont@unav.es; 4 Department of Chemistry, Faculty of Sciences, University of Navarra, Irunlarrea s/n, 31080 Pamplona, Spain; moharriz@unav.es

**Keywords:** rifampicin, cyclodextrin, inclusion complexation, characterization, molecular docking

## Abstract

Inclusion complexation of rifampicin (RIF) with several types of cyclodextrins (βCD, hydroxypropyl-βCD, γCD, hydroxypropyl-γCD) in aqueous solutions at different pH values was investigated to assess the interactions between RIF and cyclodextrins (CDs). Molecular modeling was performed to determine the possible interactions between RIF and CDs at several pH values. The inclusion complexes were characterized by differential scanning calorimetry, Fourier transform infrared spectroscopy, powder X-ray diffractometry, and scanning electron microscopy. Moreover, this study evaluated the dissolution profile and antibacterial activity of the formed complexes. Phase solubility analysis suggested the formation of RIF-CD affirmed 1:1 stoichiometry at all pH values (except RIF-βCD at pH 4.0 and both βCD and γCD at pH 9.0). The inclusion complexation of RIF with CD successfully increased the percentage of RIF released in in vitro studies. The inclusion complexes of RIF exhibited more than 60% of RIF released in 2 h which was significantly higher (*p* < 0.05) than release of pure RIF, which was only less than 10%. Antibacterial activity of RIF-CD complexes (measured by the minimum inhibitory concentration of RIF against *Staphylococcus aureus* and methicillin-resistant *Staphylococcus aureus*) was lower for both RIF-βCD and RIF-HPγCD at pH 7.0 to pure RIF suspension. In conclusion, this work reports that both βCD and γCD can be used to enhance the solubility of RIF and thus, improve the effectivity of RIF by decreasing the required daily dose of RIF for the treatment of bacterial infections.

## 1. Introduction

Tuberculosis (TB) has been considered as an infectious disease since the 1800s and, nowadays, about one-quarter of the world’s population is suffering from this airborne disease [1,2]. Through science and technology advancement, scientists have developed TB vaccines and multi-antibiotic therapy, offering new alternatives for TB eradication [2]. Combination of rifampicin (RIF), isoniazid, pyrazinamide, and ethambutol is currently the preferred main anti-TB drugs regimen [1,3]. The World Health Organization (WHO) reported that this treatment saved 58 million lives between 2000 and 2018 [2]. However, up to 28% of TB patients experience an increase in alanine aminotransferase (ALT) levels during treatment. ALT is an enzyme associated with hepatocellular injury. Hence, hepatoxicity has been reported as the most frequent adverse effect of TB treatment [4,5].

Generally, TB drugs are administered orally to be absorbed and directly carried to the liver, through splanchnic circulation, where they undergo first pass metabolism [6]. Therefore, liver activity is negatively affected by daily repeated oral dosing of TB drugs. Additionally, upper gastrointestinal bleeding has been also reported as an adverse effect, due to hemorrhagic gastric erosions, which has been associated with daily administration of RIF [7]. RIF is known to have a complex structure and poor solubility, which may reduce oral bioavailability (approximately 68% in humans) [8]. As a result, RIF requires quite large doses for oral administration (450–600 mg per day). Indeed, the length of TB treatment must be six months or longer to successfully overcome the bacterial infection. Therefore, the large doses of RIF might contribute to worsening the hepatoxicity and gastrointestinal disturbance of patients [9].

There is an urgent need of new strategies to address the adverse effects associated with the oral administration of RIF to improve the efficiency and effectiveness of TB treatment. A preferred strategy to enhance the solubility of hydrophobic drugs is by forming an inclusion complex with cyclodextrins (CDs) [10,11]. Briefly, the structure of CDs consists of a hydrophobic cavity and hydrophilic surface [12,13,14]. Inclusion complex formation of CDs involves the RIF molecule moving inside the hydrophobic cavity. Due to the large capacity of their cavity, βCD and γCD may be most suitable to accommodate the large structure of RIF. The solubilizing effect of CDs is the result of noncovalent bonding formation of the hydrophilic surface with free -OH groups of the surrounding aqueous medium [15]. Up to now, several type of CDs have been used to form inclusion complexes with RIF in order to increase its solubility, such as native βCD [16,17], hydroxypropyl-βCD [18,19], and randomly methylated-βCD [16,18]. However, to the best of our knowledge, complexation studies of RIF with native and derivatives of γCD have not previously been reported. Moreover, the comparison of native and derivatives of both βCD and γCD to form RIF inclusion complexes have not yet been described.

In the present work, the behavior of RIF-CD inclusion complexes was studied to demonstrate the usefulness of βCD and γCD in enhancing RIF solubility. Molecular modeling was conducted to determine the most favorable inclusion complex that was possible to form in stoichiometry 1:1 for CD:RIF, taking the analyzed CD as the host (receptor) and RIF as the guest (ligand). Moreover, the relative stability of different poses obtained at the selected pH values was evaluated. Then, the inclusion complexes were characterized by using differential scanning calorimetry (DSC), Fourier transformed infrared spectroscopy (FTIR), and X-ray diffraction methods. Furthermore, phase solubility, complexation constant determination, and dissolution studies were also conducted. Finally, the in vitro antibacterial activity was assessed against *Staphylococcus aureus* and methicillin-resistant *Staphylococcus aureus* (MRSA) by determining the minimum inhibitory concentrations (MIC).

## 2. Materials and Methods

### 2.1. Materials

Rifampicin (RIF) was purchased from Alfa Aesar (Lancashire, UK). Cavamax™ W7 Pharma (βCD), Cavamax™ W8 Pharma (γCD), Cavasol™ W7 HP Pharma (hydroxypropyl-βCD), Cavasol™ W8 HP Pharma (hydroxypropyl-γCD) were provided by Ashland (Kidderminster, UK). Ultrapure water was obtained from a water purification system Elga PURELAB DV 25, Veolia Water Systems (Dublin, Ireland). All other chemicals and materials were of analytical grade and purchased from Sigma-Aldrich (Dorset, UK) and Fisher Scientific (Leicestershire, UK). Phosphate buffers were prepared according to method described previously [16]. The ionic strength of buffers was 0.05 M. The pH values of buffered solutions were adjusted using phosphoric acid (purity 85%) or sodium hydroxide 0.1 N to achieve pH 4.0, 7.0, and 9.0, respectively.

### 2.2. Saturation Solubility

Solubility studies were performed to assess RIF solubility in a range of buffers (pH 4.0, pH 7.0, and pH 9.0). For this purpose, an excess amount of RIF was added to 3 mL of adjusted buffered solutions in a glass vial. Suspensions were stirred at 100 rpm in a shaker incubator (Jeio Tech ISF-7100, Medline Scientific, Chalgrove Oxon, UK) at 37 °C for 24 h. Samples were then filtered using 0.2 μm syringe filters (Minisart^®^, Sartorius Stedim Biotech, Goeltingen, Germany). All samples were diluted appropriately and analyzed using the validated HPLC method described in Section 2.10.

### 2.3. Molecular Modeling Study

The calculations were performed on SGI Virtu VS100 workstations, provided with MOE2019.1001 software package. The reference models AJUVEG and CIWMIE10 were obtained from the Cambridge Structural Database (CSD System version 5.41: search and information retrieval with ConQuest version 2.0.4, structure visualization with Mercury 4.3.0) and used as templates for building the initial models for the analyzed cyclodextrins [20,21]. The RIF 3D model was obtained from the database Cambridge Structural Database (CSD ref LOPZEX). The reference model was exported to the MOE2019.0102 work space, and the structure was carefully curated in a Born implicit solvent model (dielectric = 80), using the implemented AMBER10:EHT force field. A preliminary optimization was carried out with a root mean square gradient (RMS) of 0.1 kcal/mol/Å^2^ as completion criterion [22]. Restraints and constraints were not applied. The first minimized conformation obtained was considered as the starting point for the conformational analysis carried out through a LowmodeMD approach implemented in MOE2019.0102 suite with a rms = 0.001 and an energy window of 5 kcal. This approach generates conformations using a short ~1 ps run of Molecular Dynamics (MD) at constant temperature (300 °K) followed by an all-atom energy minimization. Once the conformational trajectory was obtained, a representative lowest energy conformation was selected. The intramolecular hydrogen bonds framework was analyzed at pH 4.0, 7.0, and 9.0. The electrostatic map was calculated for the selected representative conformation, detecting the preferred regions for hydrogen bond acceptors or positive electrostatic potential at a potential value = −2 kcal/mol and the regions for hydrogen bond donor or negative electrostatic potential at a potential value = −2 kcal/mol. The interaction potential map was calculated for the selected representative conformation, detecting the preferred regions for interaction with an OH2 probe at −5.5 kcal/mol and a dry probe at −2.5 kcal/mol. As complementary data, the mean conformational trajectory values for van der Waals volume and surface, and accessible solvent (water) surface area were calculated. With regard to the analyzed cyclodextrins, the 3D models of βCD and γCD were abstracted from the Cambridge Structural Database (Refcodes: AJUVEG and CIWMIE10 respectively), exported to the MOE workspace (AMBER10:EHT forcefield, Born implicit solvent model), and later, they were subjected to a mild minimization process (steepest descent rms = 0.1 kcal/mol/Å^2^ as completion criterion). The water molecules present in the models were eliminated. The assignment of protonation state and geometry, at the different analyzed pH was achieved by the application of the Protonate3D option implemented in the MOE2019.0102 suite [23]. The 3D HP-βCD and HP-γCD inclusion complexes were constructed, in similar conditions, from the initial AJUVEG and CIWMIE10 models, respectively, which were modified to the desired structure by Builder module implemented in the MOE2019.0102 suite. The substitution degree and the location of the 2-hydroxypropyl chain were selected according to the data obtained in recently published studies [24]. Once constructed, a mild minimization protocol (steepest descent rms = 0.1 kcal/mol/Å^2^ as completion criterion) was applied. The minimized conformation obtained was considered as the starting point for the conformational analysis carried out through a LowmodeMD approach implemented in MOE2019.0102 suite with a rms = 0.001 and an energy window of 5 kcal. Once the conformational trajectory was obtained, a representative lowest energy conformation was selected. The electrostatic map was calculated for the selected conformation, detecting the preferred regions for hydrogen bond acceptors or positive electrostatic potential at a potential value = −2 kcal/mol and the regions for hydrogen bond donor or negative electrostatic potential at a potential value = −2 kcal/mol. The interaction potential map was calculated for the selected representative conformation, detecting the preferred regions for interaction with an OH2 probe at −5.5 kcal/mol and a dry probe at −2.5 kcal/mol. The protonate 3D approach implemented in MOE2019.0102 suite was applied for the preparation of the different CDs as receptor, taking into account the selected pH (4.0, 7.0, or 9.0). RIF is prepared as a ligand, also taking into account the pH and the possible existing protomers depending on it. Two different docking strategies were applied according to the solvent conditions: implicit or explicit. The first docking study, carried out by applying the Dock algorithm implemented in the MOE2019.0102 suite (AMBER10:EHT force field, Born implicit solvent model, dielectric= 80) can be summed up as follows: (a) selection of the host (receptor) atoms in the analyzed cyclodextrin; (b) selection of the guest (ligand): RIF in the lowest energy conformation; (d) selection of the placement method: Triangle Matcher (London dG Scoring) with 100 as maximum pose number; (e) selection of refinement method: Induced Fit (GBVI/WSA dG Scoring) with 25 as maximum pose number; (f) selection of the best poses and further clustering analysis of the results. The protocol for the second docking study was similar. The explicit solvent (water) conditions were simulated by applying the solvate routine implemented in the MOE2019.0102 suite (Spherical droplet mode, NaCl 0.15 mol/L). A set of flowcharts describing the procedures followed during the molecular modeling studies can be found in Figure S1 (Supplementary Materials).

### 2.4. Phase Solubility

Phase solubility analysis was carried out similarly to the saturation solubility studies by using a range of concentrations of CDs in buffered solutions at different pH vales. CD solutions of βCD, HP-βCD, γCD, and HP-γCD were prepared with different concentrations (0, 0.5, 1, 1.5, 2.0, 2.5 mM) at pH 4.0, 7.0, and 9.0, respectively. Solubility of each drug was determined and plotted in a graph with the nominal concentration of cyclodextrin on the x-axis and the nominal concentration of RIF on the *y*-axis.

### 2.5. Constant Calculations

In order to determine the binding strength of CD and RIF, the binding constant (Ks) was calculated based on the phase diagram of solubility studies. If the results showed that complexes formed following a 1:1 stoichiometry (A_L_-type phase solubility diagram and a slope less than 1), the Ks was determined using Equation (1) [18].

(1) $$Ks=\frac{\left[RIF-CD\right]}{{\left[RIF\right]}_{free}\cdot {\left[CD\right]}_{free}}\;\;\;\;\;CD+RIF\overset{Ks}{↔}CD-RIF$$

where [*RIF − CD*]*,* [*RIF*]*_free_*, and [*CD*]*_free_* are concentration of the RIF-CD complexes, free RIF, and free CD, respectively.

[*RIF*]*_free_* can be considered as RIF initial solubility (*S*_0_), therefore Equation (2) becomes:

(2) $$Ks=\frac{\left[RIF-CD\right]}{S_{0}\cdot {\left[CD\right]}_{free}}$$

The phase solubility diagram was constructed by plotting the RIF molar concentration on the *y*-axis and the CD molar concentration on the *x*-axis. Hence, Ks was calculated using the slope of the linear regression, as shown in Equation (3).

(3) $$Ks=\frac{slope}{S_{0}\cdot \left(1-slope\right)}$$

The complexation efficiency (CE) was determined by using the slope of the phase solubility diagram according to Equation (4) [15].

(4) $$CE=\frac{\left[CD-RIF\right]}{{\left[CD\right]}_{free}}=S_{0}\cdot Ks=\frac{slope}{1-slope}$$

Based on CE determination, the CD molar ratio was calculated using Equation (5) [25].

(5) $$Drug\;:CD\;molar\;ratio=1\;:\;\frac{\left(CE+1\right)}{CE}$$

### 2.6. Preparation of RIF-CD Complexes

**Figure 1:**
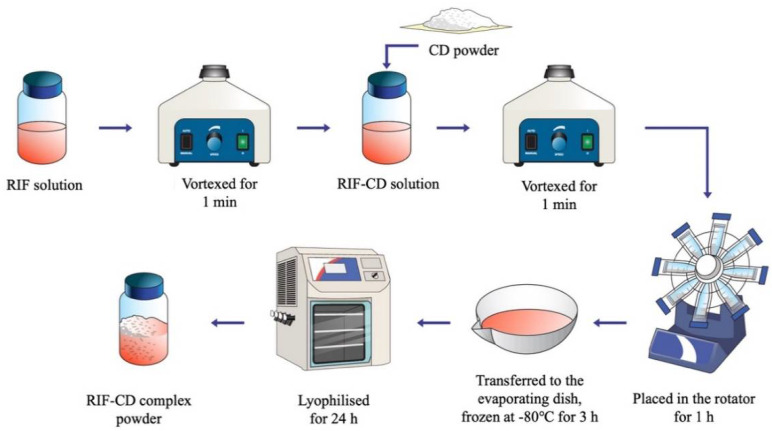
Schematic diagram of RIF-CD complex preparation.

RIF-CD inclusion complexes were obtained using a freeze-drying method, as illustrated in Figure 1. The weighed RIF powder was dissolved in 25 mL of a buffered solution at pH 4.0, 7.0, and 9.0 by homogenizing them using a vortex mixer (Fisons Scientific, Leicestershire, UK) at 3200 rpm for 1 min. Each βCD, HP-βCD, γCD, and HP-γCD powder was added to the previous RIF solutions and then vortexed again at 2000 rpm for 1 min. Following this, RIF-CD solutions were placed in a rotator (Stuart™ SB2 Rotator Disk, Fisher Scientific, Leicestershire, UK) at 20 rpm for 1 h and then frozen in a −80 °C freezer for 3 h. Subsequently, the frozen solutions were transferred to a freeze drier (Virtis™ Advantage XL-70, SP Scientific, Warminster, PA, USA) and kept for 24 h inside the vacuum chamber (50 mTorr) using the following cycle: primary drying for 13 h at a shelf temperature starting from −40 °C and secondary drying for 11 h at 25 °C [26,27].

### 2.7. Characterization of Inclusion Complexes

#### 2.7.1. Differential scanning calorimetry

DSC analysis of pure RIF, βCD, HP-βCD, γCD, HP-γCD, and all RIF-CD systems (physical mixtures and complexes) were performed using a DSC Q100 (TA Instruments, Elstree, Hertfordshire, UK). Each sample was scanned at the range 25–300 °C by increasing the heat rate of 10 °C/min under the nitrogen flow.

#### 2.7.2. FT-IR Spectroscopy

FTIR spectra of the different samples were recorded using a Spectrum Two™ instrument (Accutrac FT/IR-4100™, Perkin Elmer, Waltham, MA, USA) by the attenuated total reflectance (ATR) technique. The spectra were recorded at room temperature from 4000 to 400 cm^−1^, with a resolution of 4.0 cm^−1^ and a total of 32 scans.

#### 2.7.3. X-ray Diffraction Analysis

XRD analysis was performed using MiniFlex II Dekstop Powder X-ray Diffractometer (Rigaku Corporation, Kent, UK) equipped with Cu K_β_ radiation (λ = 1.39 Å), at a voltage of 30 kV, a current of 15 mA, and at room temperature. All samples were scanned for 2.0°/min with an angular range of 5–30° 2θ (2 theta) in continuous mode with a sampling width of 0.03°.

#### 2.7.4. Morphology and Structure

The morphologies of all samples were observed using scanning electron microscopy (SEM) (Tabletop Microscope TM3030, Hitachi, Krefeld, Germany). SEM images of samples were recorded at an accelerating voltage of 15 kV under vacuum conditions and were captured at ×250 magnification.

### 2.8. In Vitro Release Study

In vitro release profiles for pure RIF and RIF-CD inclusion complexes were evaluated at pH 7.0 and 9.0. Simulated gastric fluid (pH 1.2), simulated intestinal fluid (pH 6.8), and PBS (pH 7.4) were prepared as the release media in this study and used to mimic biological conditions in the human body [28]. All samples were added into Eppendorf tubes containing 2 mL of the release media and were then placed in an incubator at 37 °C with shaking at 40 rpm. Sampling was carried out at 1, 2, 3, 4, 5, 6, 7, 8, and 24 h by taking 200 µL of each sample, transferring them to HPLC vials containing glass inserts, and replacing the sample with 200 µL of fresh release media. The samples were analyzed using a validated HPLC method.

### 2.9. In Vitro Antibacterial Activity

The minimum inhibitory concentration (MIC) of pure RIF and RIF-CD complexes was determined against both *Staphylococcus aureus* (NCTC^®^ 10788) and methicillin-resistant *Staphylococcus aureus* (MRSA) (ATCC^®^ 33593™) using the broth microdilution method [19,29]. Briefly, inclusion complexes of RIF-CD were separately dissolved in sterile distilled water and diluted with Mueller–Hinton broth (MHB) to obtain a series of culture media containing different RIF concentrations ranging from 50 to 0.78 µg/mL. Solutions and suspensions of pure RIF were prepared by adding the pure RIF in methanol and in sterile distilled water, respectively, and then diluted with MHB to achieve the same concentration as for the RIF-CD inclusion complexes. Culture media in the microplates were inoculated with 100 µL of each bacterial suspension to achieve a final bacterial concentration of 2.0 × 10^5^ CFU/mL. Pure MHB inoculated with the different bacterial strains was included as a positive control. The inoculated culture media were then incubated at 37 °C for 24 h. The MIC was determined as the lowest concentration of pure RIF and RIF-CD inclusion complexes where no visible bacterial growth was detected.

### 2.10. Instrumentation and Chromatographic Conditions for RIF Quantification

The HPLC method for RIF quantification was adapted from our previous work [26]. HPLC Agilent 1220 Series (Agilent Technologies, UK Ltd., Stockport, UK) was used to analyze all the samples. Isocratic separation was achieved using a Phenomenex^®^ Luna C_18_ (ODS1) column (150 × 4.6 mm internal diameter, 5 µm packing) (Phenomenex, Cheshire, UK). The injection volume was 50 µL and the analysis was carried out at room temperature with a flow rate of 1 mL/min. The mobile phase was a mixture of 25 mM sodium dihydrogen phosphate buffer (with 1% triethylamine and adjusted using phosphoric acid at pH 6.8) and methanol (ratio 30:70 *v*/*v*). RIF was detected by a UV at 334 nm. All chromatograms were analyzed using the Agilent ChemStation^®^ Software B.02.01.

### 2.11. Statistical Analysis

Statistical analyses were performed using GraphPad Prism^®^ version 8.0 (GraphPad Software, San Diego, CA, USA). One-way analysis of variance (ANOVA) was used to compare the results and assess whether there were significant differences between the means of data sets. In all cases, *p* < 0.05 was used to denote statistical significance, where *p*-value outputs were 0.033 (*), 0.002 (**), and <0.001 (***).

## 3. Results and Discussion

### 3.1. Saturation Solubility

**Figure 2:**
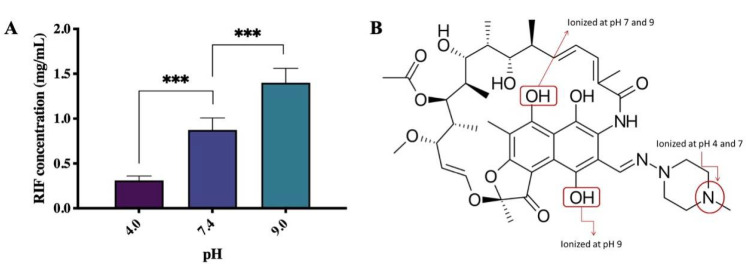
(**A**) RIF solubility at a range of buffer pH values (means + SD, *n* = 12) and (**B**) RIF structure showing the ionizable groups. Differences were calculated using one-way ANOVA, Tukey’s post hoc test, and deemed significant at where *p*-value outputs were 0.033 (*), 0.002 (**), <0.001 (***) and <0.0001 (****).

The results of RIF saturation solubility studies in a range of buffered solutions are presented in Figure 2A. RIF possesses a solubility of 0.31, 0.87, and 1.40 mg/mL at pH 4.0, 7.0, and 9.0, respectively. At pH 7.0, the solubility of RIF was three times higher than at pH 4.0 and almost two times lower than at pH 9.0 (*p* < 0.05). Therefore, it can be seen that the solubility of RIF can be categorized as pH dependent. These results agreed with those previously discussed [18,30]. RIF has a broad variation of solubility at different pH values due to its zwitter-ionic properties given by its complex molecular structure, leading to the formation of several ionizable species by changing the pH of the aqueous medium. The three ionizable groups between pH 4 and 9 are the hydroxyl groups and the 4′-piperazin nitrogen (Figure 2B). The three ionic species that can be expected according to the pKa values are a cationic form predominating at pH before 1.7, a globally neutral zwitterionic form between pH 1.7 and 7.9 and an anionic form above 7.9. At pH 9, the predominant species is a dianionic one. Lower solubility at pH 4.0 might be due to the zwitterionic form of RIF. The zwitterionic form may then be replaced by the anionic form of RIF as the pH is increased, resulting in an enhanced solubility of RIF at pH 7.0 and 9.0 [18].

### 3.2. Molecular Modeling Studies

**Figure 3:**
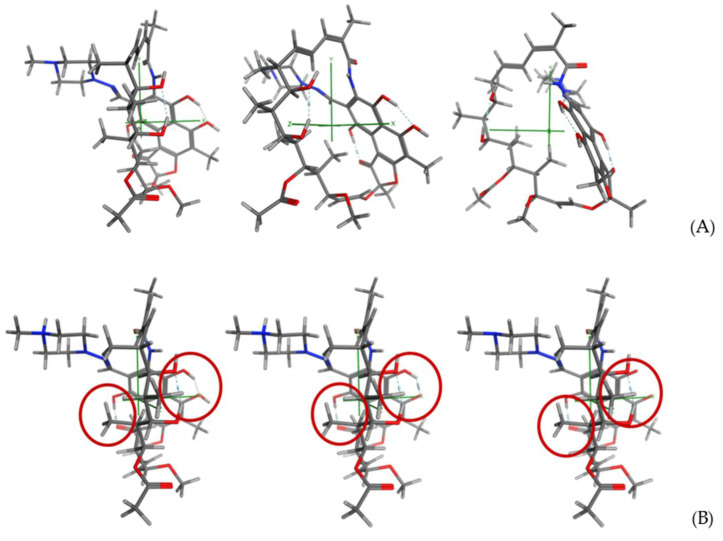
(**A**) 3D model for RIF showing the molecular axis. (**B**) Intramolecular hydrogen bonds framework (in red) for RIF at pH 4.0, 7.0, and 9.0 (lowest energy conformation).

Regarding the RIF structure, it can be considered as a large, bulky fragment, consisting of the central ring of the structure, with a side chain containing the piperazine ring, linked by means of an imine moiety. The mean van der Waals volume is 787.23 Å3, the mean van der Waals surface area is 833.98 Å2, and the solvent (water) accessible surface area, ASA, is 1004.12 Å2. The presence of a naphthalene ring decorated with hydroxyl groups, together with the carbon chain that forms the core ring, preforms three zones of different polarity: (a) the hydrophobic naphthalene pi-ring; (b) the hydrophobic zone in which the sp2 and sp3 carbon atoms are found without further decorations; and (c) a more polar zone decorated by ester and hydroxyl groups. The preliminary analysis regarding its conformational behavior shows a preferred lowest energy conformation, according to which piperazine ring is projected outward the bulky core moiety. The intramolecular framework of hydrogen bonds contributes to the stabilization of this conformation (Figure 3). Table S1 (Supplementary Materials) shows the corresponding electrostatic and interaction potential maps obtained at the different pH values. As it can be seen, and predictably, the interaction profile of the molecule is directly related to the gradual emergence of the different protomers depending on the pH.

Regarding the βCD model, once the initial conformation was constructed, a minimization protocol was applied and the selected lowest energy conformation was analyzed in order to obtain information regarding both the acceptor and donor hydrogen bonds and hydrophobic profiles of the inner cavity, the receptor in which, according to our working hypothesis, RIF will be accommodated. The data obtained (Table S2, Supplementary Materials) shows an area of considerable size that is distributed along the edges of the minor face; this area forms as a wall of hydrogen bridge donors which is constructed of the hydroxyl groups located at position 6 of the rings. Only small surfaces related with acceptor features are distributed along the external surface. In the inner cavity a discontinuous area where the inwardly oriented OH moieties act as donors is detected, whereas a continuous hydrophobic region is located in the center of this cavity.

In relation with the interaction map carried out with a OH2 probe (see Table S2, Supplementary Materials) the data obtained, which can be related with the solvation profile, shows how, in addition to the outer areas of the molecule that may be attractive to solvent molecules, there is a distribution of solvent accessible areas along the inner wall, which surround the central hydrophobic area of the inner cavity.

With respect to γCD, the 3D model construction and the selection of the representative lowest energy conformation, follow the same protocol described for βCD. The data obtained regarding the distribution of donor, acceptor, hydrophobic area features, and the interaction with the selected OH2 probe (Table S2, Supplementary Materials) shows a similar behavior to that obtained for βCD, although a wider acceptor zone is detected in the outer surface of the structure. Moreover, the inner hydrophobic area is larger but less continuous than for βCD.

**Figure 4:**
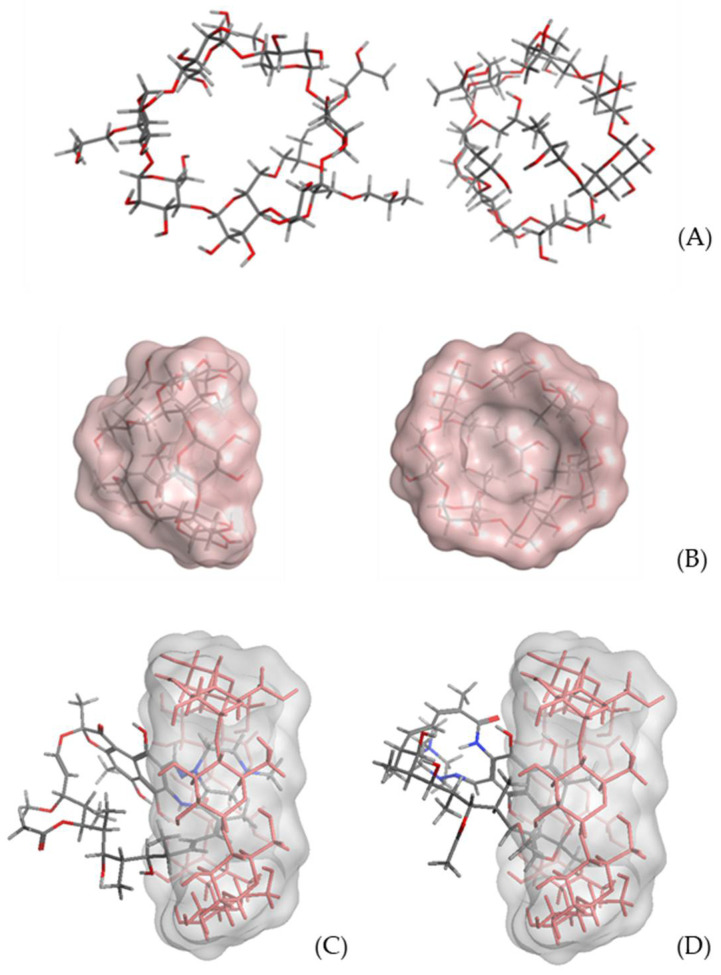
(**A**) Open (left) and closed (right) representative lowest energy conformation for 2HP-βCD (sticks, C = grey; H = white; O = red). (**B**) Lateral and frontal (mayor face) perspective for closed conformation (molecular surface). Representative best poses for RIF-ɣCD complexes. (**C**) N-(4-methylpiperazin-1-yl)methanimine moiety placed in the inner cavity. (**D**) Bezo[f]benzofuran-3-one moiety placed in the inner cavity.

With respect to the derivatized cyclodextrins, HP-βCD and HP-γCD, the presence of the 2-hydroxypropyl chain substituted on the minor face, makes it necessary to carry out a study of their conformational behavior, regarding the final spatial distribution of these side chains. Once the representative lowest energy conformation was obtained for each of the derivatized cyclodextrins, HP-βCD and HP-γCD, following the same protocol applied for the βCD and γCD models, the conformational analysis was carried out through a LowmodeMD approach implemented in MOE2019.0102. Once the conformational trajectory was obtained, the obtained conformations were clustered taking as separation criteria the energy range. This way, two representative lowest energy conformations were selected for each derivatized CD (Figure 4A). In the first of them, both in HP-βCD and HP-γCD, it is observed that the side chains are directed towards the outside of the surface of the structure (open conformation), while in the second one the chains are projected towards the interior of the minor face (closed conformation). The closed conformation, with lower energy in both cases, is stabilized by a network of hydrogen bonds. The shape of the structure is now as a cone (Figure 4B), with the vertex consisting of the chains that cover the minor face. Taking into account the aforementioned data, we selected the closed conformation, both for HP-βCD and HP-γCD ones, as receptor models for the corresponding docking studies.

The acceptor and donor hydrogen bonds and hydrophobic profiles, as well as the solvation profile for derivatized cyclodextrins, are shown in Table S3 (Supplementary Materials). As can be observed, the presence of a 2-hydroxypropyl moiety in the minor face alters these profiles, as compared with the native cyclodextrins, especially regarding the acceptor and hydrophobic areas. In fact, the inner hydrophobic region has an irregular distribution, and the access through the minor face to the inner cavity seems slightly blocked.

The first docking analysis was carried out applying an implicit solvent (water) strategy and taking the lowest energy conformation of the corresponding CD as a rigid receptor, whereas RIF was taken as flexible ligand. Protonation states of receptor and ligand were achieved by means of Protonate 3D approach (see experimental section for details). The poses obtained were clustered according to their final energy (Table S2, Supplementary Materials). The second docking analysis was carried out applying an explicit solvent strategy, by applying a solvate routine with spherical droplet mode (water) and NaCl 0.15 mol/L. In this case, as in the previous approach, RIF is taken as a flexible ligand and the corresponding CD as a rigid receptor. Protonation state and clustering are achieved in a similar way. In a first approximation, a greater ability to complex with γCD is observed (Tables S4–S6, Supplementary Materials).

Taking as a reference the score values of the cluster that groups the best poses obtained, it can be deduced that the formation of RIF-CD complexes is favored when the receptor is γCD; thus, in the complexes obtained in the implicit solvent approach, it is observed that, given the size of the inner cavity of this CD, it can house both the N-(4-methylpiperazin-1-yl)methanimine moiety and the benzo[f]benzofuran-3-one fragment (Figure 4C,D).

Concerning βCD, the preferred poses show the N-(4-methylpiperazin-1-yl)methanimine fragment strongly anchored in the internal cavity, with a significant influence of the pH, since it is observed that the stability of the complex increases, as the pH increases. The data obtained in the explicit solvent approach show a similar behavior for both βCD and γCD complexes, with greater stability for complexes with γCD. With regard to modified HP-βCD and γCD cyclodextrins, the data obtained allowed us to suspect that the piperazine side chain of RIF is preferably enclosed into HP-βCD hydrophobic cavity, whereas for HP-γCD the complex shows the bulkiest region of RIF molecule is preferably enclosed into cyclodextrin cavity. In fact, the results indicate that the most stable complex (due to less energy value) has the RIF complexed with HP-βCD through the piperazine tail enclosed into the hydrophobic cavity of HP-βCD and the bulky region of the molecule in the surface cavity of cyclodextrin [16,19,31]. The data regarding HP-γCD shows that the preferred poses have the bulky fragment of RIF placed in the inner cavity.

Table S8 (Supplementary Materials) shows the models for the studies carried out, in which the RIF structures chosen as representatives of each of the clusters obtained, represented in sticks, are superimposed, and the CD used in each case.

### 3.3. Phase Solubility Study

**Figure 5:**
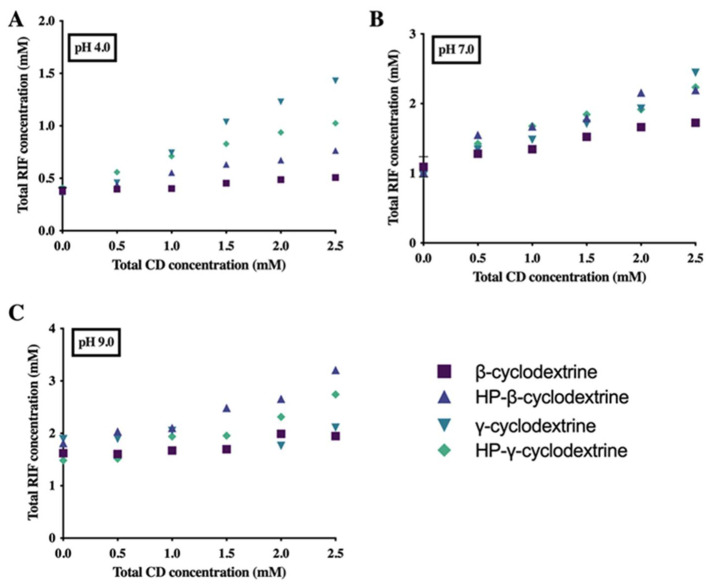
Phase solubility diagrams of RIF with βCD, HP-βCD, γCD, and HP-γCD in (**A**) pH 4.0, (**B**) pH 7.0, and (**C**) pH 9.0.

The phase solubility profiles obtained from the complex formation between RIF and native and derivative CD (βCD, HP-βCD, γCD, and HP-γCD) in buffered solutions at different pH values are presented in Figure 5. The results showed that the solubility of RIF increased linearly with CD concentration at pH 4.0 and 7.0 in all cases. On the other hand, at pH 9.0 linear increase of drug solubility is mainly detected for complexes with derivative CDs (RIF-HP-βCD and RIF-HP-γCD). The solubility diagram type observed was a linear one (A_L_), which indicates a 1:1 complex formation (slope lower than 1). At pH 9.0, RIF solubility does not increase with modified CDs. This could be associated with the way RIF is accommodated in the CD cavities described in the molecular modeling section. Moreover, the ionization of the drug in this media will contribute to this behavior.

### 3.4. Determination of Constant Values: Stability Constant, Complexation Efficiency, and Solubility Enhancement Ratio

The Ks values obtained from the slope of the linear phase diagrams are presented in Table 1. It is worth nothing that in both pH 4 and 7, a high increase of Ks values is detected for γCD complexes with respect to βCD. This fact would be in accordance with the modeling studies data above described that postulate the formation of CD complexes favored when the receptor is γCD as consequence of the larger inner cavity of this CD. In addition, the derivative β-CD complexes show greater stability constant values than those formulated with the native form at both pH values. It could be associated with the distorted cavity shape due to the presence of hydroxypropyl chains on the surface of CD which could facilitate the drug inclusion, as previously mentioned. Inclusion behavior of RIF in both native and derivative γCD differs from that found with βCD. At pH 4 and 7, Ks values for HPγCD complexes are lower than those corresponding to its native CD related to how the RIF molecule is hosted. The molecular modeling studies show that the bulky fragment of RIF could be placed in the inner cavity and, as a consequence of a certain steric hindrance, the stability constant decreases. In addition, taking into account the pH influence on γCD complexation, a decrease in the Ks value is observed at pH 7 with respect to pH 4 for γCD and especially for HPγCD systems (2510 versus 1284 M^−1^). This could be explained by two different factors. First of all, the ionization of RIF. Finally, the fact that the bulkiest part of drug molecule is enclosed to the CD cavity will contribute to this behavior.

At pH 7, the RIF-βCD and RIF-HPβCD complexes were found to be more stable compared to at pH 4. These results are in accordance with the well-known fact that the neutral form of a molecule leads to a more stable inclusion complex because of its hydrophobicity and so its higher affinity to the hydrophobic cavity of CD [31].

However, the presence of the di-anionic form of rifampicin at pH 9 could hinder its inclusion in the cavity of the native cyclodextrins and, therefore, it is only possible to determine the stability constant in the modified cyclodextrins in which the inclusion is favored as result of the structure of the cavity. In addition to the stability constant, Loftsson et al. have previously stated that CE can be used to assess drug solubility enhancement by CD in a more reliable fashion [25]. CE is calculated based on the slope of the phase solubility diagram only, without considering S0 and y-intercept. For instance, inclusion complexes of RIF-HPβCD at pH 4.0 had a CE of 0.20 (1:6 molar ratio), indicating that, in acidic solutions, only 1 out of 6 CD molecules in solution are forming water-soluble complexes with each RIF molecule at any instant. Alternatively, for βCD (1:12 molar ratio) twice as many CD molecules are required to achieve the same effect. Thus, only 1 out of 12 of these CD molecules are forming complexes with 1 RIF molecule at any one time. Again, this is due to the smaller cavity size of βCD.

Increasing pH to 7.0 resulted in an increase of the CE. Two CD molecules per drug molecule are necessary for drug inclusion complexation at these pH values for RIF-HPβCD, RIF-γCD, and RIF-HPγCD. Nevertheless, for βCD complexes, twice as many molecules of this type of CD are required to solubilize a single RIF molecule (molar ratio 1:4). This result is in line with previously reported results due to the cavity size for βCD [32,33].

The molecular modeling results included in Section 3.2 showed that the piperazine ring of RIF is entrapped into the hydrophobic CD cavity to form an inclusion complex. Therefore, partial ionization (between pH 4.0 and 7.0) is preferable to form RIF-CD complexes, as the 3-piperazin nitrogen groups are not fully ionized at this particular pH. Therefore, RIF is still able to interact with -OH groups in the hydrophobic cavity of CD.

### 3.5. Characterization of Inclusion Complexes

#### 3.5.1. Differential Scanning Calorimetry

**Figure 6:**
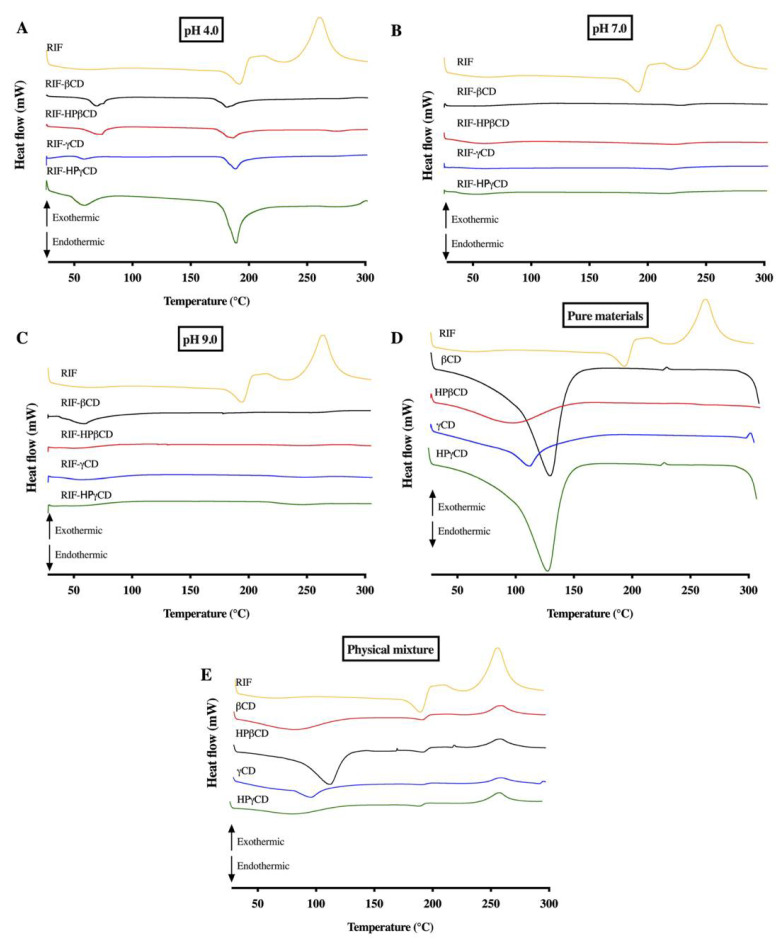
DSC thermograms of RIF-CD complexes with several types of CD at (**A**) pH 4.0, (**B**) pH 7.0, and (**C**) pH 9.0. The thermograms of RIF-CD complexes compared to thermogram of (**D**) pure RIF, pure CD, and (**E**) physical mixture. Exothermic is up and endothermic is down.

**Figure 7:**
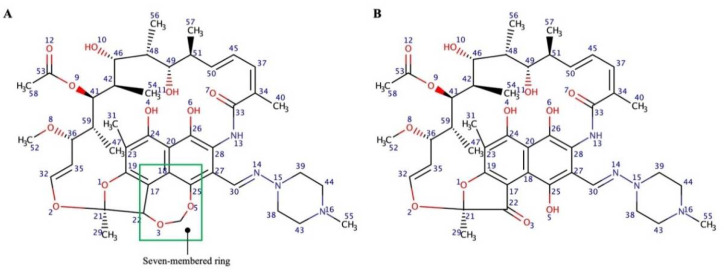
Chemical structure of RIF polymorphism in (**A**) form I and (**B**) form II.

Pure RIF, CDs, RIF-CD physical mixtures, and RIF-CD inclusion complexes were characterized using DSC in order to study drug inclusion with CDs. DSC thermograms (Figure 6) of pure RIF showed a sharp endothermic peak at 191 °C and two exothermic signals at around 205 and 259 °C, corresponding to the melting point of the crystalline form II of this drug. This peak was followed by a recrystallization to form I and its decomposition. These results are in concordance with those reported by Alves et al. and Agrawal et al. who analyzed the different thermal behavior of the two crystalline forms, namely form I and form II [30,34]. In the physical mixture thermograms, the dehydration process of CD followed by the thermal behavior of RIF previously analyzed can be detected. A higher aqueous solubility was found in form I in comparison to form II due to a strong hydrogen bond between C25 hydroxyl and furanone forming a seven-membered ring and acting as a lone pair donor (Figure 7) [35]. Contrastingly, in form II, C25 hydroxyl does not form a bond with the furanone, hence, a seven-membered ring is not formed [35]. As a result, form II is less polar and less soluble than form I, requiring a higher energy to be decomposed. In the first endothermic peak, form II underwent a melting process, followed by recrystallisation (exothermic peak) [36].

The disappearance of endothermic fusion peak of RIF (form II) and both exothermic events, transition (form II to form I) and decomposition of polymorph I for all RIF-CD complexes evidences the inclusion of RIF molecules into the CD cavities at both pH 7 and 9. However, the thermal behavior of inclusion complexes at pH 4 shows the endothermic melting peak of RIF (191 °C) shifted to 188 °C for all CDs tested. This fact could be associated to a potential partial inclusion of RIF molecule in the CD cavity. Nevertheless, this endothermic peak was entirely missing when the complexes were formed at pH 7 and 9. Therefore, it can inferred that both neutral and basic solution are preferable to form the RIF-CD complexes, as has been previously reported [18,34].

#### 3.5.2. FT-IR Spectroscopy

**Figure 8:**
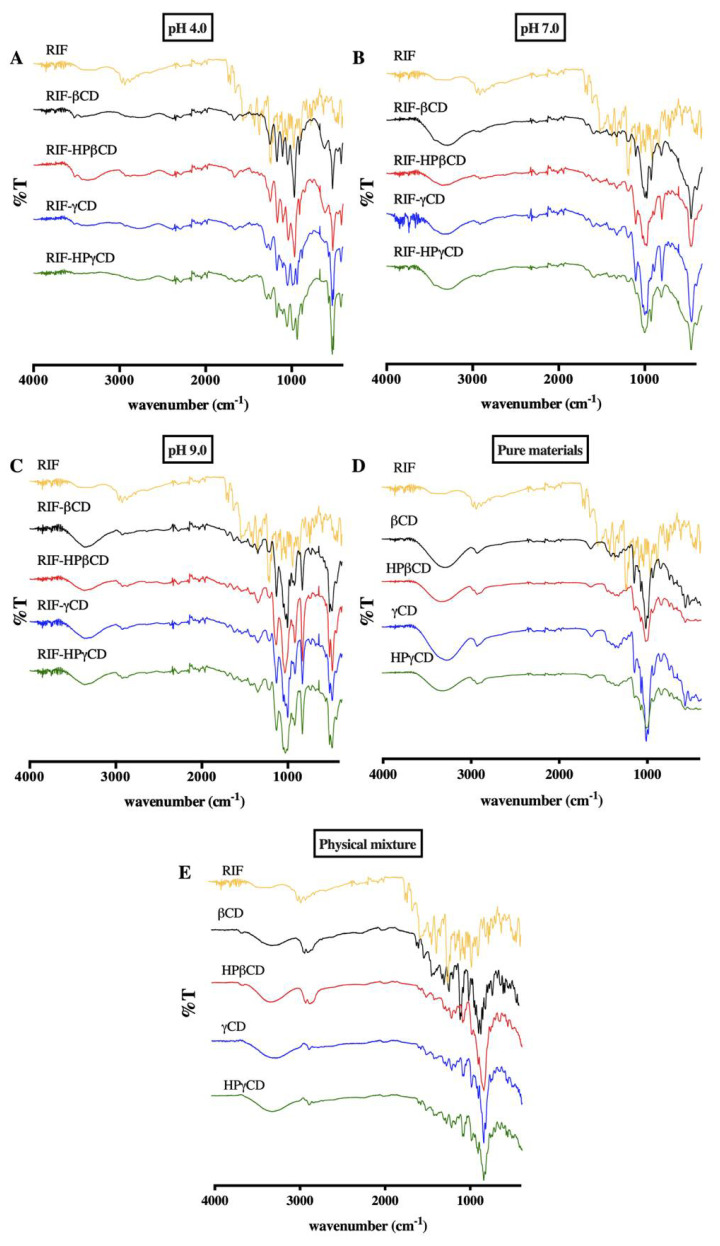
Representative FTIR spectra of RIF and several types of CD at (**A**) pH 4.0, (**B**) pH 7.0, and (**C**) pH 9.0. (**D**) The IR spectra of pure RIF, pure CD, and (**E**) physical mixture of RIF and CD.

RIF-CDs samples were characterized using ATR-FTIR spectroscopy in order to obtain further supporting evidence on the formation of inclusion complexes. The infrared spectrum (Figure 8) of pure RIF showed the presence of several peaks at 3524–3629 cm^−1^ (free -OH stretching vibration), 2768 cm^−1^ (stretching vibration of H-C=O:C-H band), double peak at around 1706 cm^−1^ (stretching vibration of C=O band) and 1643 cm^−1^ (C=N stretching vibration of piperazine ring). In addition, the characteristic double peak (at around 1706 cm^−1^) is associated with RIF form II. It can be assigned to the stretching vibrations of acetyl and furanone C=O bonds [30]. Moreover, the broad band over 3500 cm^−1^ (OH band) confirms the presence of RIF form II as reported previously on the DSC analysis section [30]. It is notable that the presence of a broad band at 3200–3300 cm^−1^, where there is O-H stretching, represents the intermolecular bond formed in all complexes. This peak indicates that RIF, as a guest molecule, successfully entered and bonded to OH groups in the CD hydrophobic cavity. The bands corresponding to the C=O group were clearly reduced in intensity for the CD complexes. Moreover, a peak at 1643 cm^−1^, which corresponds to C=N stretching in the piperazine ring of RIF, was not detected in all spectra of RIF-CD complexes. This might be attributed to the piperazine group of RIF being included in the cavity of CD, as previously described in Section 3.2. Hence, spectra obtained from FTIR studies confirmed the results obtained by DSC. All peaks were found to be similar compared to the other RIF-CD complexes at the same pH of complexation medium. Slight shifts or changes in spectra of RIF-CD complexes at different pH values might be caused by different interactions between RIF or CD in the buffered solution [37].

#### 3.5.3. X-ray Diffraction Analysis

**Figure 9:**
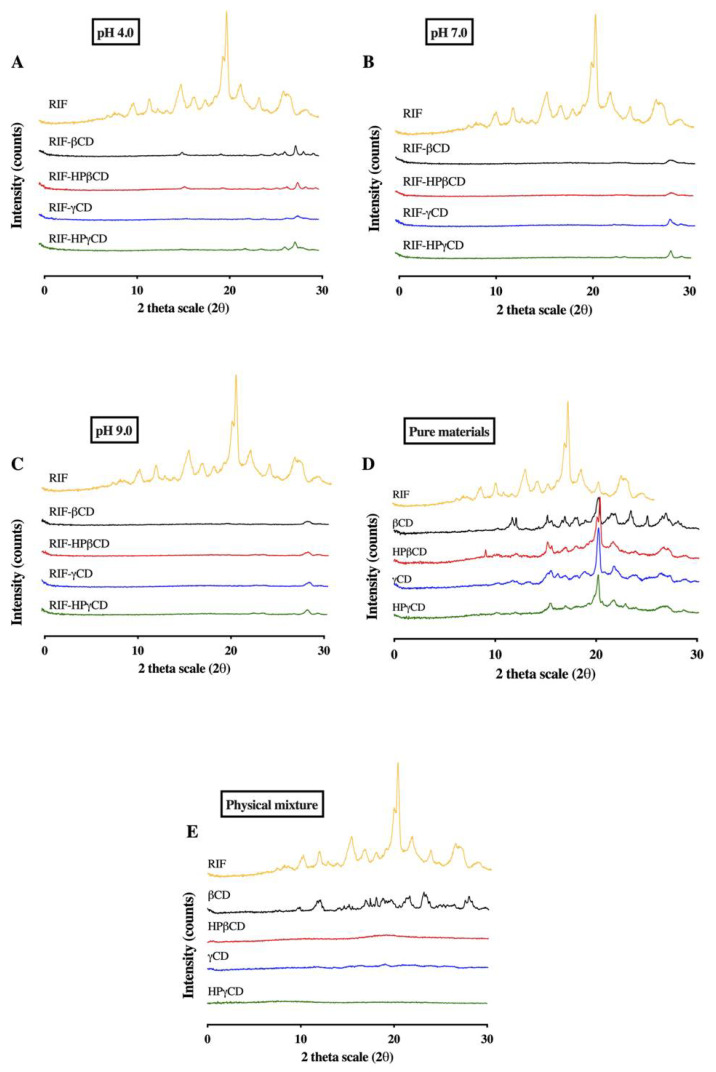
Representative of XRD diffractograms of RIF and several types of CD at (**A**) pH 4.0, (**B**) pH 7.0, and (**C**) pH 9.0. (**D**) The diffractograms of pure RIF, pure CD, and (**E**) physical mixture of RIF and CD.

The diffraction patterns of RIF-CD systems provide information about the crystalline structure of complexes. XRD diffractograms of pure RIF and CD, as well as RIF-CD physical mixtures and inclusion complexes, are presented in Figure 9. The results of pure CDs showed that the βCD presents a crystalline structure, as sharp peaks appeared in its diffractogram, whereas the other CDs showed diffuse patterns (halos) typical of amorphous structures. These findings are in agreement with those found in a previous study, where it was described that βCD presented a stable crystal molecular arrangement due to the rigid formation of hydrogen bonds between two neighboring hydroxyl groups located in the second and third glucose units [38]. This bond, a secondary belt-like structure, affects βCD molecules’ flexibility required to form intermolecular bonds with water molecules nearby, hence, reducing its aqueous solubility [10].

The crystallinity of RIF-CD complexes was determined by comparing the diffraction patterns of RIF-CD inclusion complexes with those corresponding to pure RIF and physical mixtures taken as references. It is well known that the diffraction patterns of complexes must be clearly distinct from that of the physical mixtures if the inclusion complexation process has been performed. The diffractogram for pure RIF displayed several sharp peaks at the following diffraction angles (2θ) of 12.2°, 13.7°, 16.7°, 18.1°, 19.1°, 21.1°, 22.5°, 24.3°, 27.0°, and 29.0°, indicating that RIF is presented in a crystalline form (form II), as previously showed with the DSC results. The diffractograms of physical mixtures showed a superimposition of the characteristic peaks of pure components forming the sample. Diffraction patterns of all the complexes showed the disappearance of all sharp RIF peaks, thus providing evidence for drug–carrier interaction by inclusion.

#### 3.5.4. Morphology and Structure

**Figure 10:**
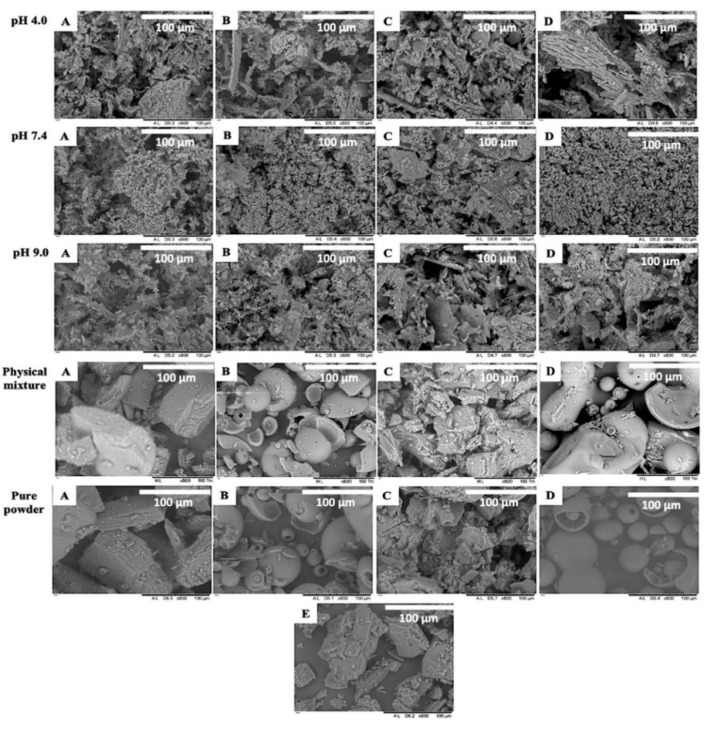
SEM imaging of RIF-CD complexes with (**A**) βCD, (**B**) HP-βCD, (**C**) γCD, and (**D**) HP-γCD compared to physical mixture, pure CD, and (**E**) pure RIF powder. The complexes were formed at different pH values of aqueous media, which are pH 4.0, 7.0, and 9.0.

Pure RIF, pure CD, and RIF-CD inclusion complexes were observed using SEM to determine their morphological and structural features (Figure 10). The results of SEM imaging confirmed the crystallinity and formation of complexes, as previously shown by DSC, FTIR, and XRD results. Pure RIF, native β-CD, and native γCD appear as plate-like crystals, whilst pure hydroxypropyl derivates of CD are presented as almost spherical particles. The SEM images of all RIF-CD inclusion complexes show a dramatic change in the morphology and structure of the complexes formed. It was no longer possible to distinguish between the pure RIF particles and the pure CD components, as a result of RIF being included in the CD system, unlike in the physical mixtures, where both components can be distinguished. This corroborated the results obtained in the DSC and XRD analyses, suggesting that RIF-CD inclusion complexes are amorphous. Moreover, it can be seen that the particle size of the inclusion complexes is smaller than the particle size of pure RIF and CDs. This parameter can influence the dissolution kinetics of the samples.

### 3.6. Dissolution Study

**Figure 11:**
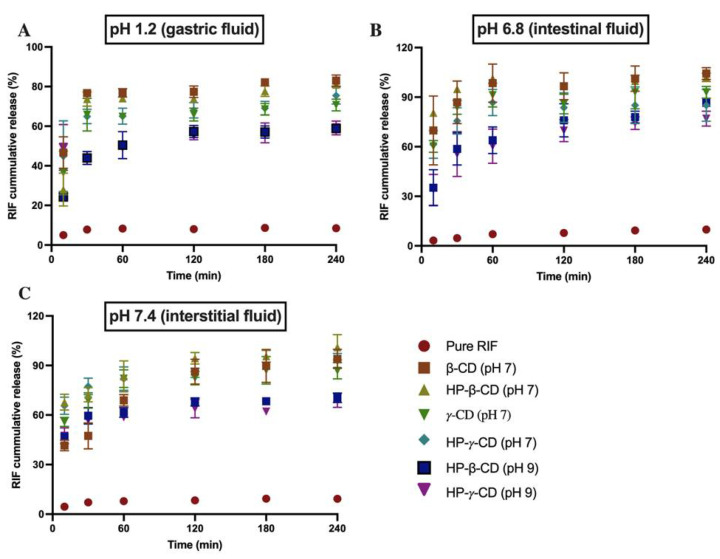
Dissolution profile of pure RIF and inclusion complexes in different pH of release media, (**A**) pH 1.2 or gastric fluid, (**B**) pH 6.8 or intestinal fluid, and (**C**) pH 7.4 or interstitial fluid (means ± SD, *n* = 3).

Dissolution profiles of pure RIF and inclusion complexes were assessed at pH 1.2, 6.8, and 7.4, simulating gastric, intestinal, and interstitial fluids. Only selected inclusion complexes, RIF-βCD, RIF-HP-βCD, RIF-γCD, and RIF-HP-γCD at pH 7.0, and RIF-HP-βCD and RIF-HP-γCD at pH 9.0 based on the result of drug content evaluation (data not shown), were assessed for this analysis. The dissolution rate profiles of pure RIF and inclusion complexes are displayed in Figure 11. The release profiles of the complexes showed that more than 60% RIF was released in 2 h in all the studied pH media compared to less than 10% for pure RIF. It is interesting to note that inclusion complexes of RIF-β-CD and RIF-HP-β-CD (pH 7) showed the highest drug release in all the studied pH media, followed by RIF-γCD and RIF-HP-γCD at pH 7.0, and RIF-HPβ-CD and RIF-HP- γ-CD at pH 9.0. This result indicates that drug release from the inclusion complex was strongly affected by the pH and the type of CD.

### 3.7. Antibacterial Activity

The MIC values of pure RIF and inclusion complexes against *S. aureus* and MRSA are presented in Table 2. The results indicated that all the samples were effective against both *S. aureus* and MRSA bacteria. Suspensions of pure RIF exhibited higher MIC values due to the lower content of RIF dissolved in the media in comparison to the drug solution. This result demonstrates that MIC of the antibacterial agent was strongly affected by the solubility. Importantly, inclusion complexes of RIF-β-CD (pH 7.0) and RIF-HP-γCD (pH 7.0) were lower than pure RIF in a suspension form, indicating that complexation of RIF and CD has improved its antibacterial activity by increasing its solubility. Moreover, the MIC values obtained for these two types of inclusion complexes is equivalent to the MIC obtained for pure RIF dissolved in methanol. It can be also seen that inclusion complexes of RIF and CD had bacteriostatic activities against *S. aureus* and MRSA, suggesting that the complexation process did not change the bacteriostatic activity of pure RIF. As previously reported by He et al. and Tewes et al., there was a significant improvement in the antibacterial activity (*p* < 0.05) of RIF encapsulation with randomly methylated β-CD and RIF-HP- βCD [18,19]. Thus, these results highlight the potential of RIF-CD complexes to maintain RIF antibacterial activity.

## 4. Conclusions

This work evaluated the inclusion of RIF into the cavity of native and derivative CDs and the effect of media with different pH values on the complexation process. The complexation of RIF with βCD, HP-βCD, γCD, and HP-γCD were found to be more effective at pH 4.0 and 7.0 based on the value of Ks and CE. The results of molecular modeling are in a good agreement with the obtained experimental data, confirming that RIF included into CD cavity which enhances RIF solubility. Moreover, the complex formation was confirmed by the characterization using DSC, FTIR, XRD. and SEM. The dissolution rate of all the inclusion complexes was much higher than that of pure RIF. Moreover, the dissolution rate was strongly affected by the type of CD and the release media used. Inclusion complexes maintained similar or higher antibacterial activity against *S. aureus* and MRSA compared with the suspension of pure RIF. Therefore, the complexation of RIF with either βCD or γCD has potential to improve RIF solubility for tuberculosis treatment.

## Figures and Tables

**Table 1:** Phase solubility data: slope and intercept, binding constant (Ks), complexation efficiency (CE), and rifampicin:cyclodextrin (RIF:CD) molar ratio.

pH	CD Type	Type of Diagram	R^2^	Intercept	Slope	Ks (M^−1^)	CE	RIF:CD Molar Ratio
4.0	βCD	A_L_	0.978	0.400	0.079	215	0.09	1:12
	HP-βCD	A_L_	0.983	0.363	0.164	541	0.20	1:6
	γCD	A_L_	0.983	0.494	0.485	2510	0.80	1:2
	HP-γCD	A_L_	0.991	0.440	0.245	738	0.32	1:4
7.0	βCD	A_L_	0.978	1.171	0.227	250	0.29	1:4
	HP-βCD	A_L_	0.961	1.096	0.480	848	0.92	1:2
	γCD	A_L_	0.965	0.950	0.549	1284	1.22	1:2
	HP-γCD	A_L_	0.959	1.145	0.435	674	0.77	1:2
9.0	βCD		-	-	-	-	-	-
	HP-βCD	A_L_	0.942	1.724	0.528	649	1.12	1:2
	γCD		-	-	-	-	-	-
	HP-γCD	A_L_	0.9366	1.370	0.497	722	0.99	1:2

**Table 2:** MIC values of pure RIF and RIF-CD complexes (*n* = 3).

Sample	MIC (µg/mL)	
	*Staphylococcus aureus* (NCTC^®^ 10788)	Methicillin-Resistant *Staphylococcus aureus* (ATCC^®^ 33593™)
Pure RIF (solution)	0.78	0.78
Pure RIF (suspension)	1.56	1.56
RIF-β-CD (pH 7.0)	0.78	0.78
RIF-HP-β-CD (pH 7.0)	1.56	1.56
RIF-γCD (pH 7.0)	1.56	1.56
RIF-HP-γCD (pH 7.0)	0.78	0.78
RIF-HP-β-CD (pH 9.0)	1.56	1.56
RIF-HP-γCD (pH 9.0)	1.56	1.56

## Data Availability

Data is contained within the article.

## Supplementary Materials

The following are available online at https://www.mdpi.com/article/10.3390/ph15010020/s1, Figure S1: Flowchart of the molecular modelling study carried out; Table S1: RIF electrostatic and interaction potential maps at pH = 4.0, 7.0 and 9.0 values (lowest energy conformation); Table S2: βCD and γCD electrostatic and interaction potential maps; Table S3: HP-βCD and HP-γCD (closed conformation) electrostatic and interaction potential maps; Table S4: Poses ranked according to the mean energy value obtained from the corresponding cluster; Table S5: Best poses obtained after docking, under implicit and explicit solvent conditions, for βCD and γCD; Table S6: Best posesa obtained after docking, under implicit and explicit solvent conditions, for HP-βCD and HP-γCD; Table S7: Overlay of representative poses after docking: βCD as receptor; γCD as receptor; Table S8: Overlay of representative poses after docking: HP-βCD as receptorc and HP-γCD as receptor.
