# Supplementary material for: Inclusion Complexes of Rifampicin with Native and Derivatized Cyclodextrins: In Silico Modeling, Formulation, and Characterization
Source: Pharmaceuticals (Basel). 2021 Dec 24;15(1):20. doi: 10.3390/ph15010020 (PMC8781390; doi:10.3390/ph15010020)
Supplement: Supplementary file 1 [file pharmaceuticals-15-00020-s001.zip › pharmaceuticals-1517477-supplementary.pdf]

Supplementary data

Inclusion complexes of rifampicin with native and derivatized cyclodextrins: *in silico* modelling, formulation and characterization.

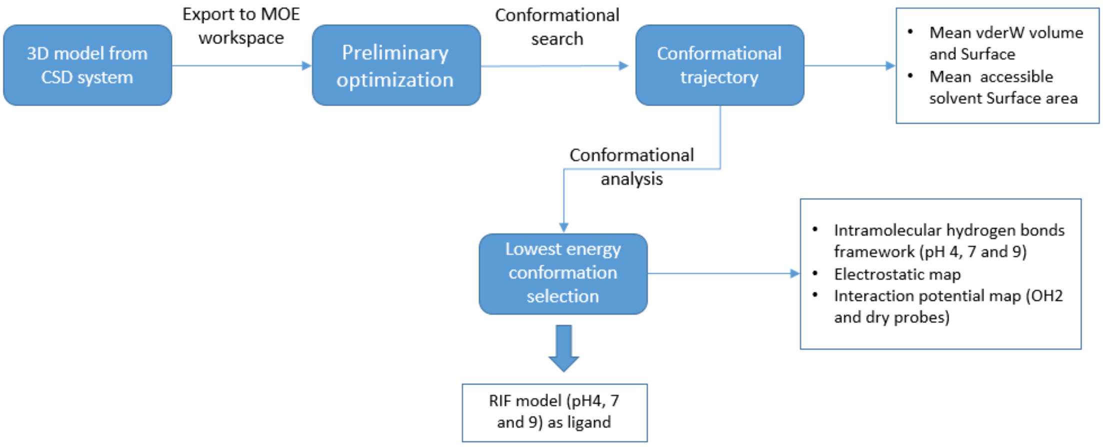

(A)

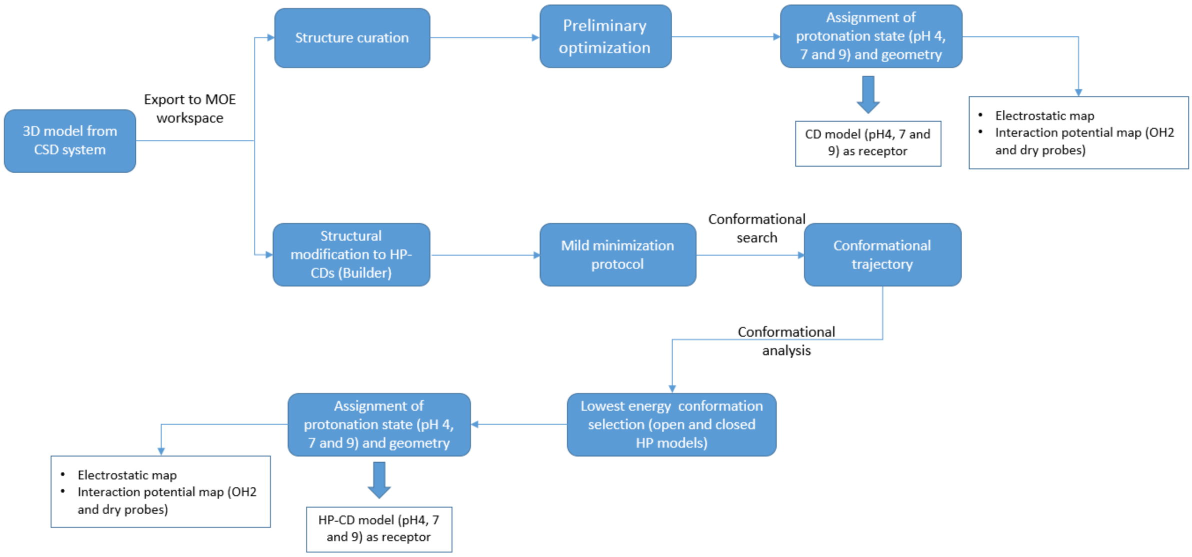

(B)

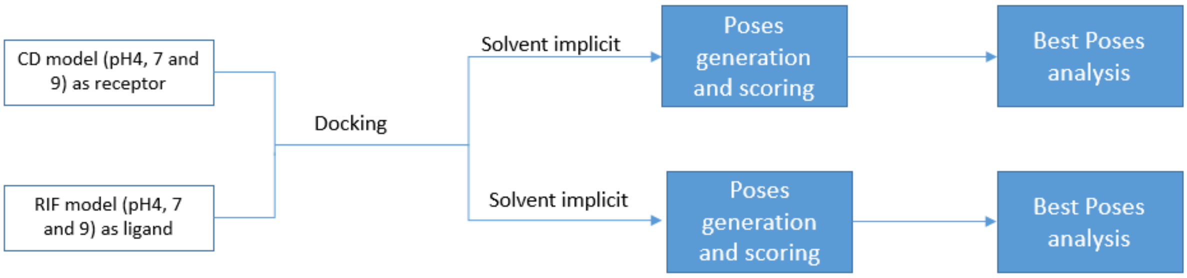

(C)

Figure S1: Flowchart of the molecular modelling study carried out: (A) RIF as ligand. (B) CD as receptor. (C) Docking

Table S1. RIF electrostatic and interaction potential maps at pH= 4.0, 7.0 and 9.0 values (lowest energy conformation).

**(a)**	4.0	7.0	9.0
**(b)**	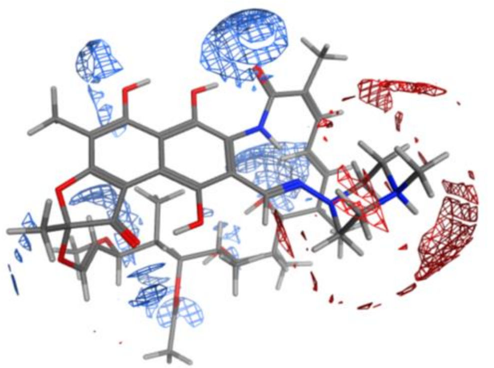	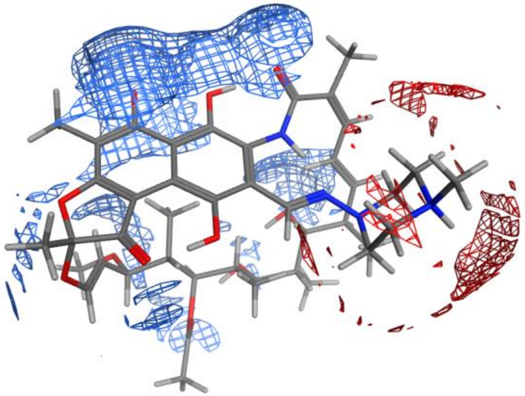	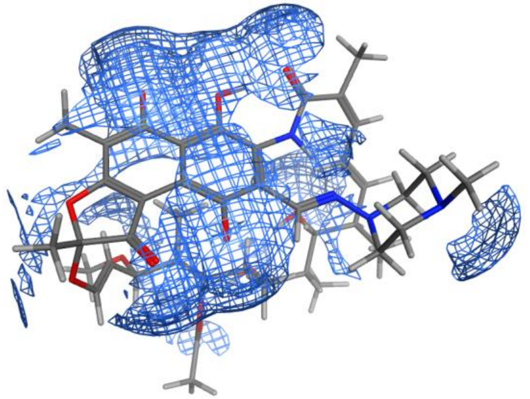
**(c)**	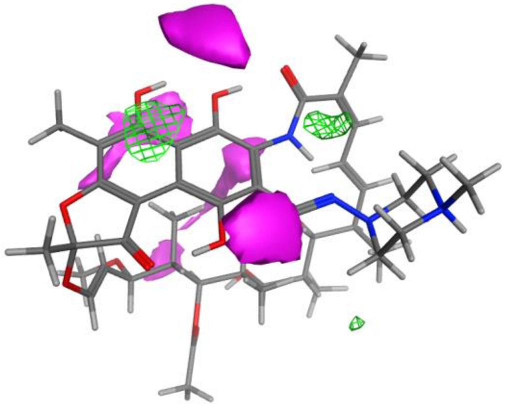	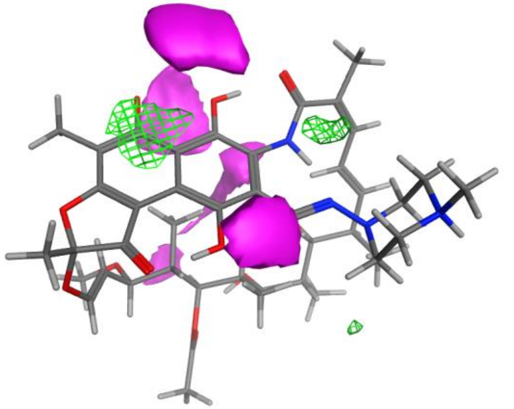	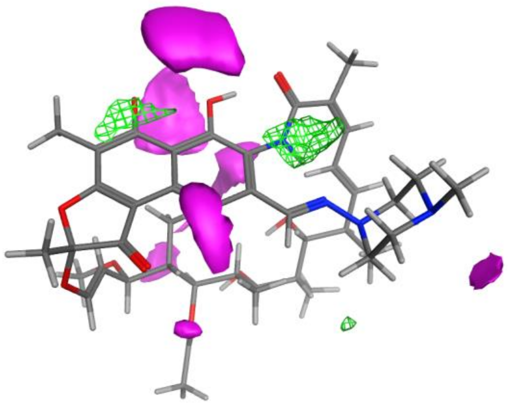

(a) pH values. (b) Electrostatic map: red line: regions for hydrogen bond acceptors or positive electrostatic potential at a potential value = -2 kcal/mol; blue line: regions for hydrogen bond donor or negative electrostatic potential at a potential value =-2 kcal/mol. (c) Interaction potential map: magenta solid: with an OH2 probe at -5.5 kcal/mol; green line: with a dry probe at -2.5 kcal/mol.

Table S2. (a) βCD and (b) γCD electrostatic and interaction potential maps.

(a)

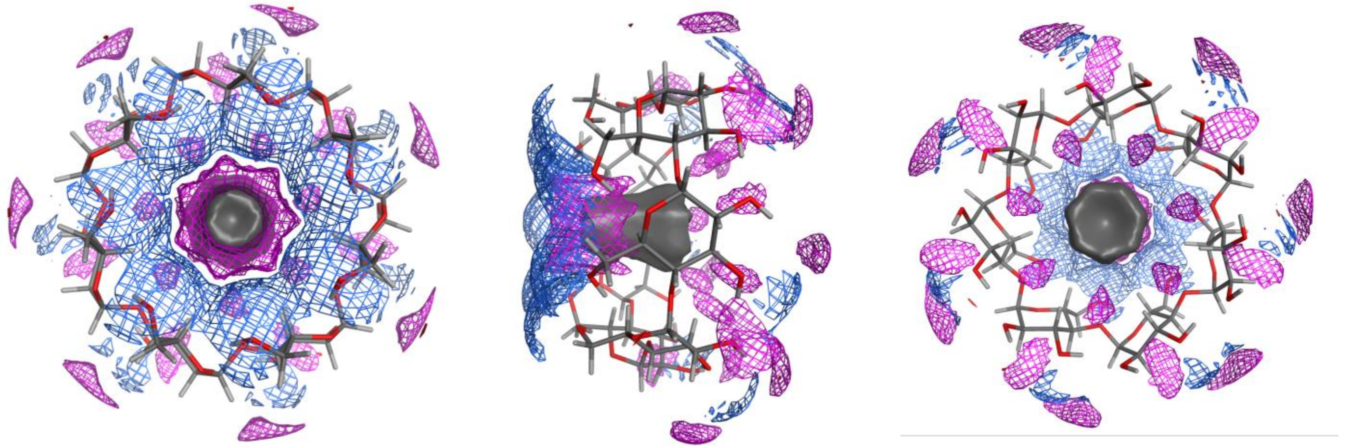

(b)

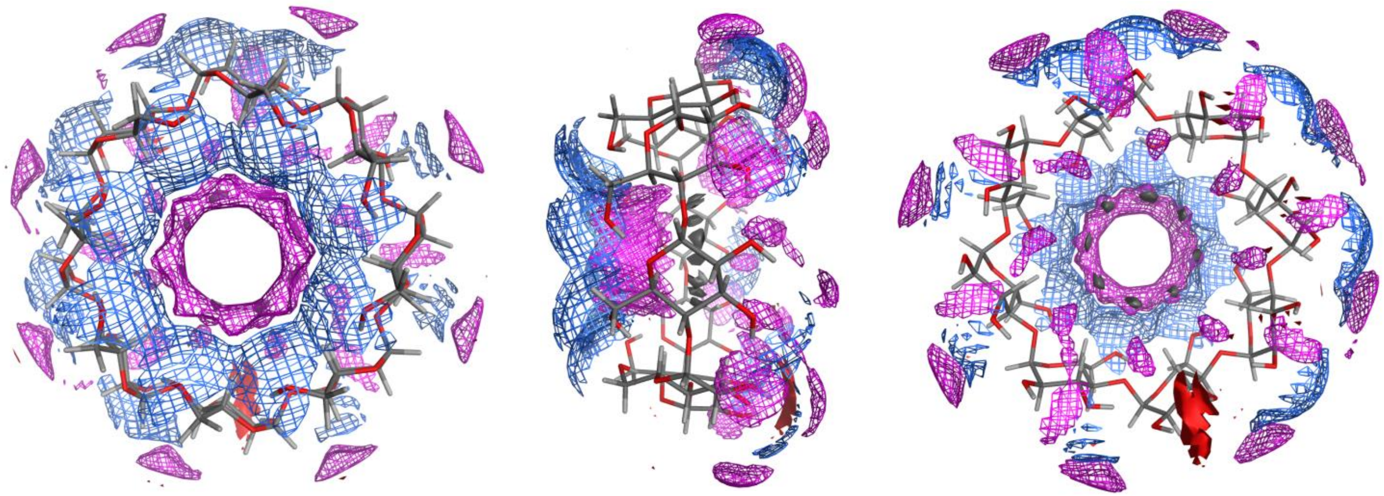

Minor face	lateral	Mayor face

(a) red solid: regions for hydrogen bond acceptors or positive electrostatic potential at a potential value = -2 kcal/mol; blue line: regions for hydrogen bond donor or negative electrostatic potential at a potential value =-2 kcal/mol. Gray solid: hydrophobic region at a potential value = -2.4 kcal/mol (b) magenta line interaction potential with an OH2 probe at -5.5 kcal/mol.

Table S3. (a) HP-βCD and (b) HP-γCD (closed conformation) electrostatic and interaction potential maps.

(a)

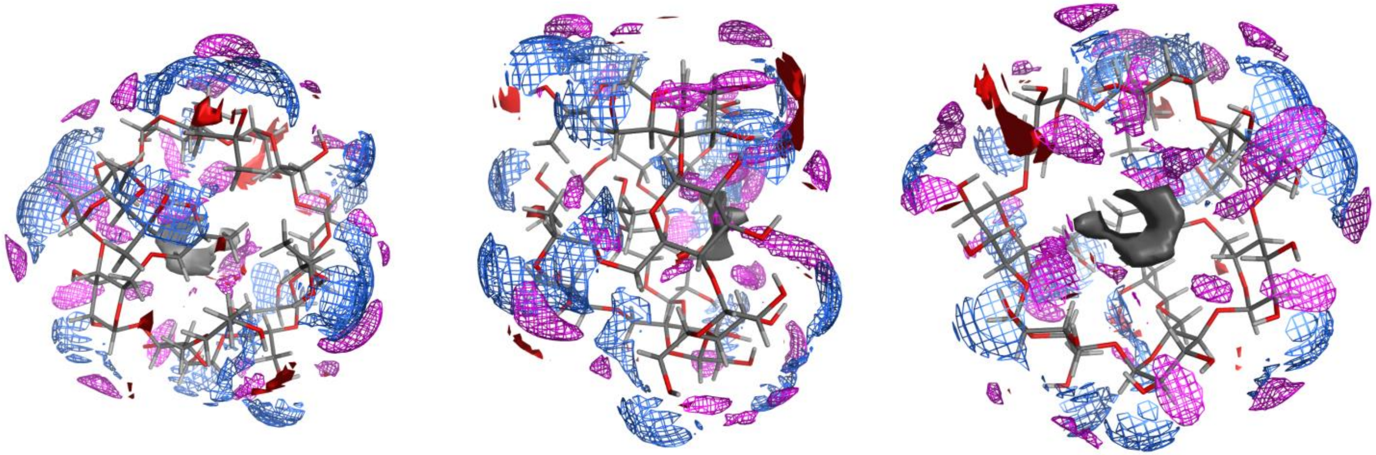

(b)

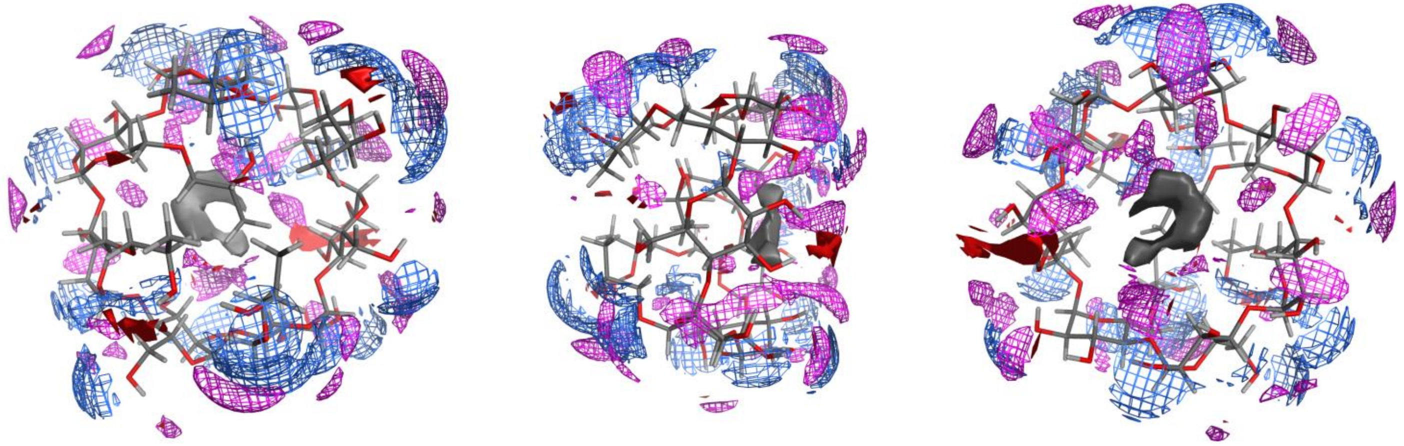

Minor face lateral Major face

(a) red solid: regions for hydrogen bond acceptors or positive electrostatic potential at a potential value = -2 kcal/mol; blue line: regions for hydrogen bond donor or negative electrostatic potential at a potential value =-2 kcal/mol. Gray solid hydrophobic region at a potential value = -2.4 kcal/mol (b) magenta line interaction potential with an OH2 probe at -5.5 kcal/mol.

Table S4. Poses ranked according to the mean energy value obtained from the corresponding cluster.

docking A[a]												
	**βCD**			**γCD**			**HP-βCD**			**HP-γCD**		
**Cluster Nº**	**pH**= 4.0	**pH**= 7.0	**pH**= 9.0	**pH**= 4.0	**pH**= 7.0	**pH**= 9.0	**pH**= 4.0	**pH**= 7.0	**pH**= 9.0	**pH**= 4.0	**pH**= 7.0	**pH**= 9.0
**1**	-6.2344	-6.9581	-7.1147	-7.4692	-7.6571	-7.6221	-7.5458	-7.2806	-7.0295	-7.5895	-7.2993	-7.6213
**2**	-5.9699	-6.7205	-6.7437	-7.4625	-7.6304	-7.1390	-7.4090	-6.6058	-6.8428	-7.4853	-7.0913	-7.2782
**3**	-5.7987	-6.6315	-6.7169	-7.0042	-7.5215	-7.0000	-7.1551	-6.5939	-6.7007	-7.2839	-6.9248	-7.1487
**4**	-5.6173	-6.6098	-6.7147	-6.8494	-7.0646	-6.9950	-6.7770	-6.5397	-6.6139	-7.2272	-6.8532	-7.1318
**5**	-5.4078	-6.5395	-6.6591	-6.7796	-7.0006	-6.9430	-6.7651	-6.5343	-6.6133	-7.0736	-6.8260	-6.9883
**docking B[b]**												

Cluster Nº	βCD			γCD			HP-βCD			HP-γCD		
	**pH=** 4.0	**pH=** 7.0	**pH=** 9.0	**pH=** 4.0	**pH=** 4.0	**pH=** 7.0	**pH=** 9.0	**pH=** 4.0	**pH=** 4.0	**pH=** 7.0	**pH=** 9.0	**pH=** 4.0
**1**	-11.5994	-11.0781	-12.5875	-12.9353	-13.1517	-15.0092	-11.4409	-12.0927	-13.5431	-12.5990	-9.9575	-11.8483
**2**	-11.4161	-10.8181	-12.5300	-12.7782	-11.8720	-14.2732	-11.0933	-12.0618	-13.4893	-12.1706	-9.5107	-11.7765
**3**	-11.3067	-10.7367	-12.1477	-12.4100	-11.5550	-14.2062	-11.0853	-11.7925	-13.1869	-11.4737	-9.4891	-11.6454
**4**	-11.2668	-10.7141	-11.9095	-12.1450	-10.6859	-14.1374	-10.9361	-11.7282	-12.8925	-11.1892	-9.0442	-11.5423
**5**	-10.9872	-10.5254	-11.3402	-12.0454	-4.0844	-13.9981	-10.8165	-11.6963	-12.6441	-11.1830	-8.8934	-11.4362

[a] Implicit solvent (water) approach. [b] Explicit solvent (water) approach

Table S5. Best poses obtained after docking, under implicit and explicit solvent conditions, for βCD and γCD.

	**implicit solvent**		**explicit solvent**	
	**βCD**	**γCD**	**βCD**	**γCD**
**pH**= 4.0	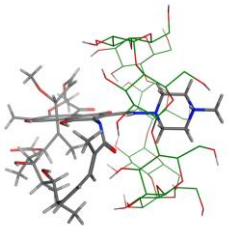	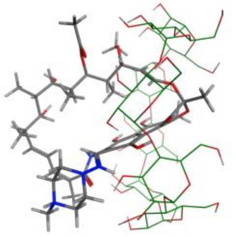	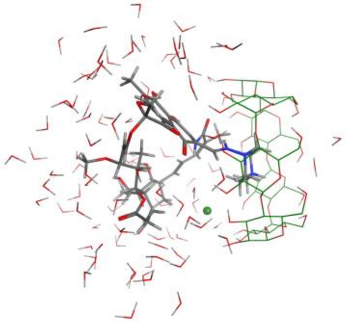	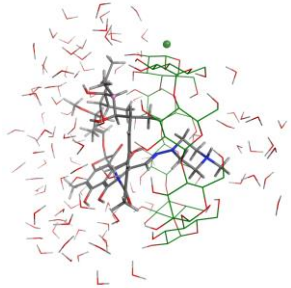
**E** $_{pose}$=	-6.234	-7.469	-11.600	-12.940
**pH**= 7.0	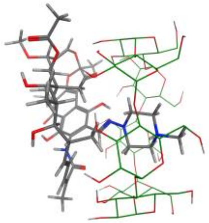	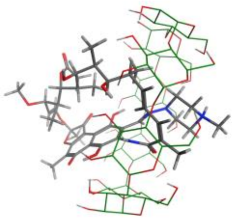	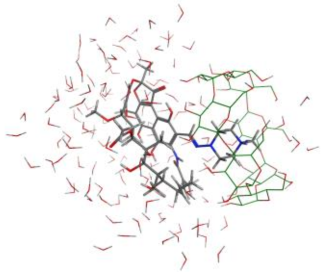	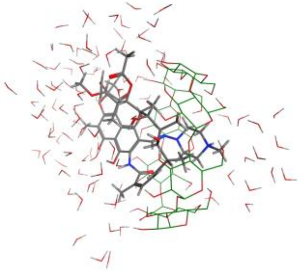
**E** $_{pose}$=	-6.958	-7.657	-11.080	-13.150
**pH**= 9.0	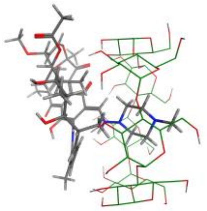	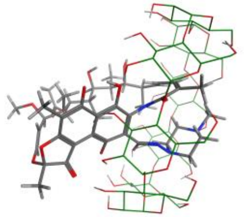	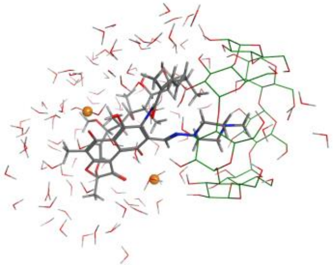	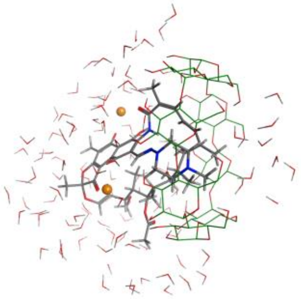

**E** $_{pose}$=	-7.115	-7.622	-12.590	-15.010

[a] RP (ligand) in sticks, C= gray; H= white; N= blue; O= red; Cyclodextrin (receptor) in sticks, C= green; O= red; solvent (water). H= white; O= red; NaCl (Salt) in balls; Na= orange; Cl= green.

Table S6. Best poses[a] obtained after docking, under implicit and explicit solvent conditions, for HP-βCD and HP-γCD.

	**implicit solvent**		**explicit solvent**	
	**2hp_βCD**	**2hp_γCD**	**2hp_βCD**	**2hp_γCD**
**pH**= 4	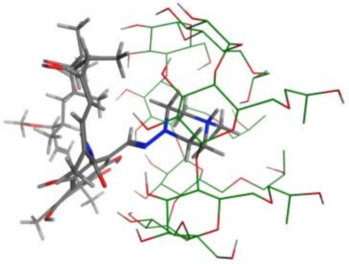	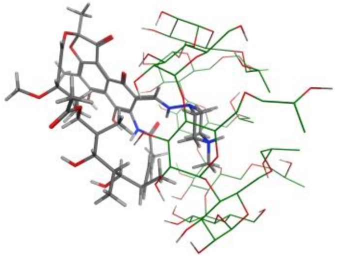	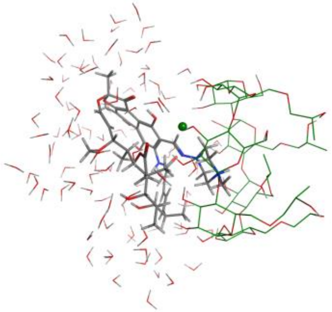	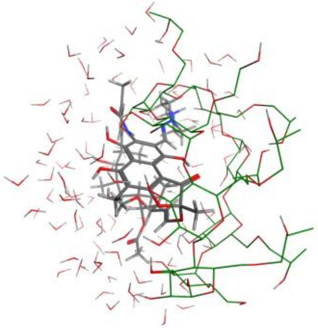
**E** $_{pose}$=	-7.546	-7.589	-11.440	-12.600
**pH**= 7	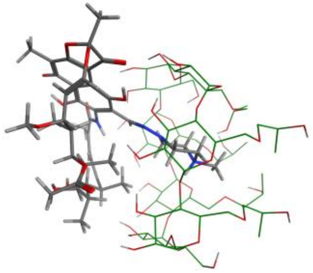	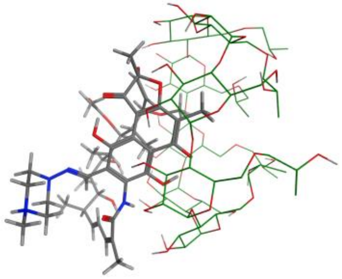	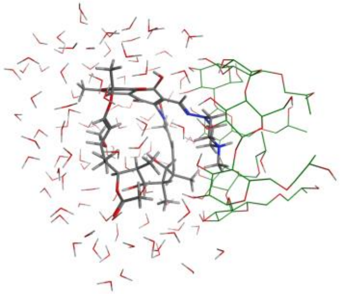	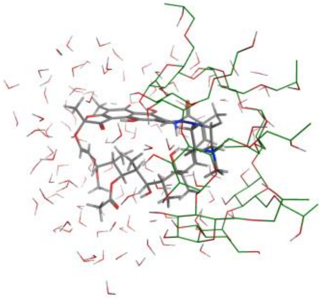
**E** $_{pose}$=	-7.281	-7.299	-12.090	-9.957

**pH=** 9	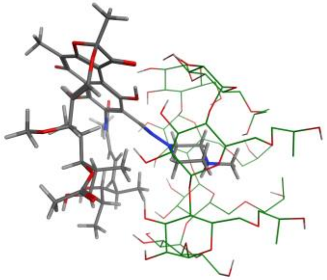	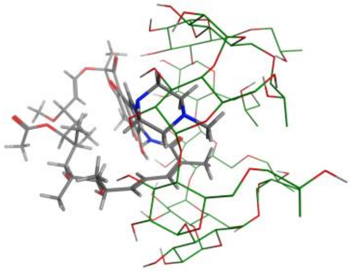	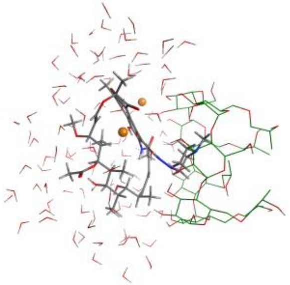	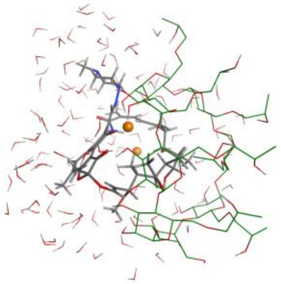
**E** $_{pose}$=	-7.029	-7.621	-13.540	-11.850

[a] RP (ligand) in sticks, C= gray; H= white; N= blue; O= red; Cyclodextrin (receptor) in sticks, C= green; O= red; solvent (water) in sticks. H= white; O= red; NaCl (Salt) in balls; Na= orange; Cl= green.

Table S7. Overlay of representative poses[a] after docking[b]: (a) βCD as receptor; (b) γCD as receptor.

	**pH** =4	**pH**= 7	**pH**= 9
**(a)**	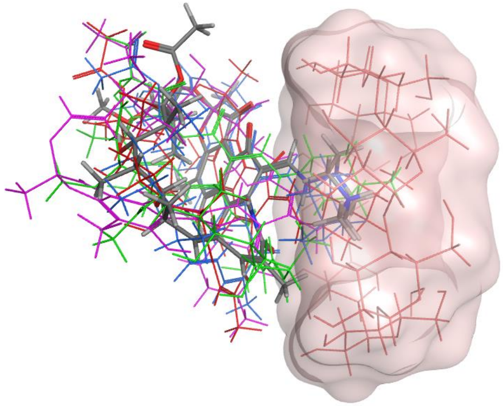	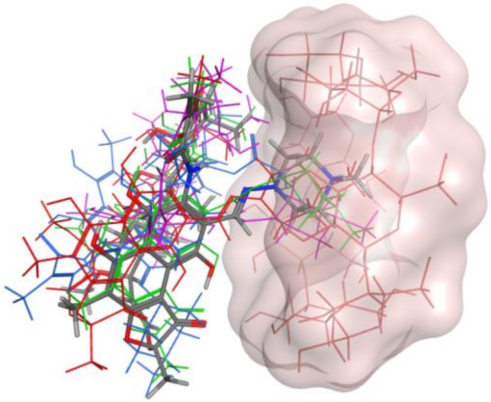	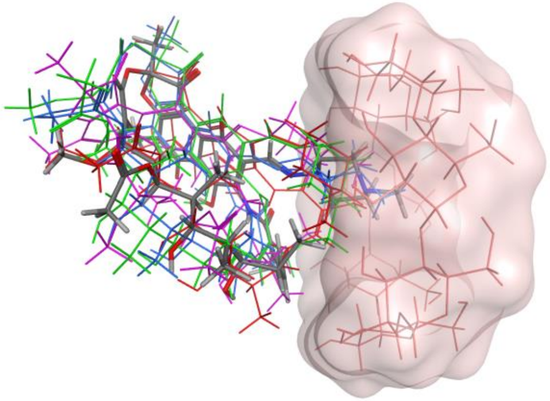
**(b)**	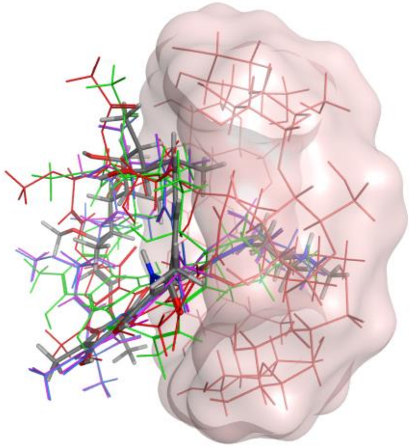	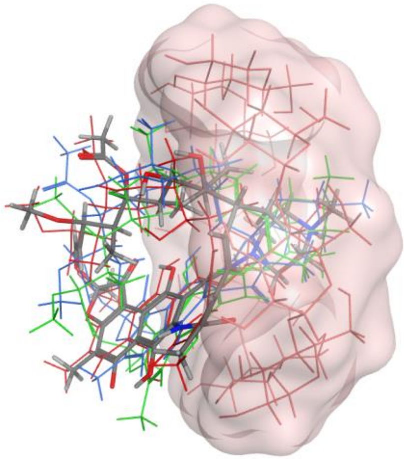	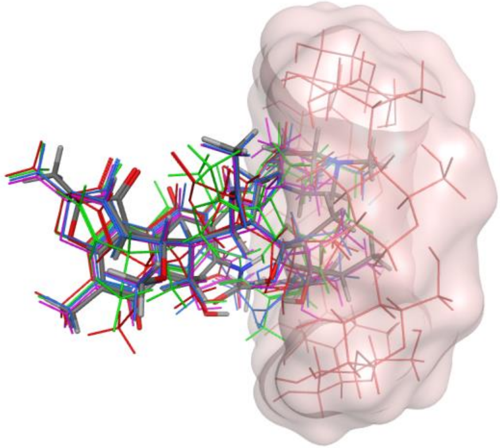

[a] Best pose representative conformation displayed as solid sticks (C= gray; H= white; N= blue; O= red). [b] Explicit solvent approach.

Table S8. Overlay of representative poses[a] after docking[b]: (a) HP-βCD as receptor[c]; (b) HP-γCD as receptor[c]

	**pH** =4.0	**pH**= 7.0	**pH**= 9.0
**(a)**	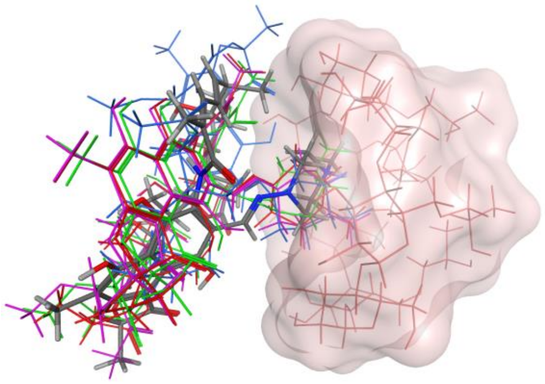	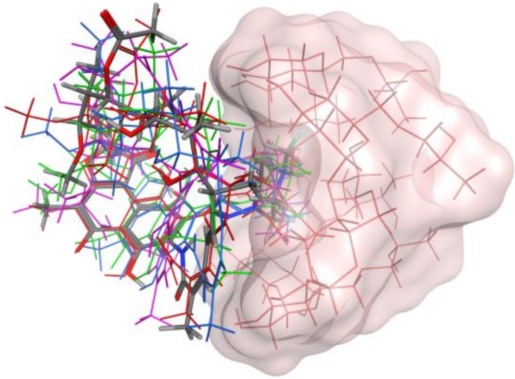	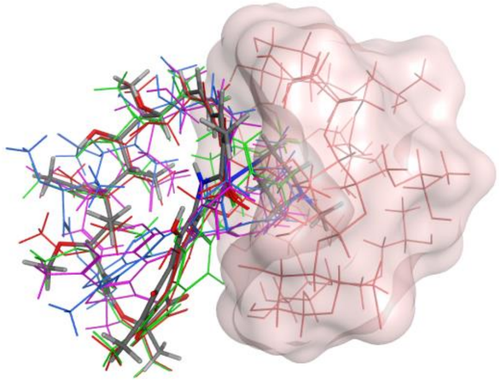
**(b)**	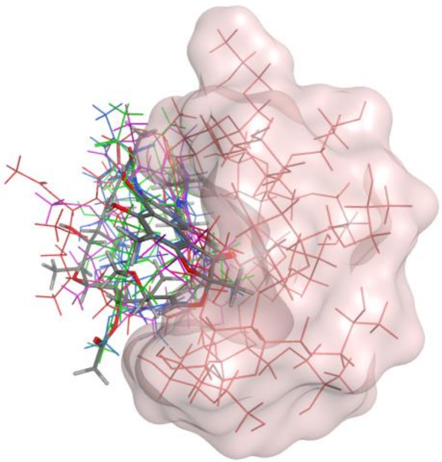	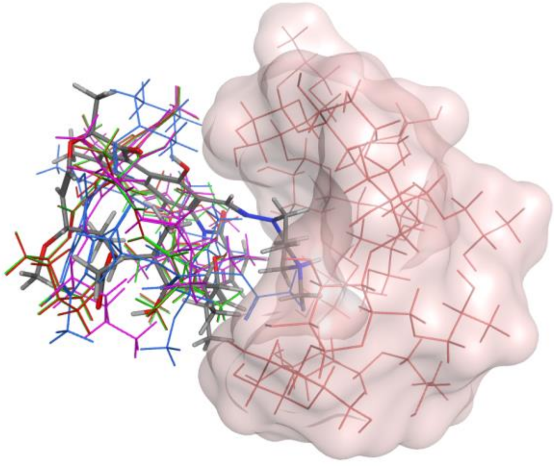	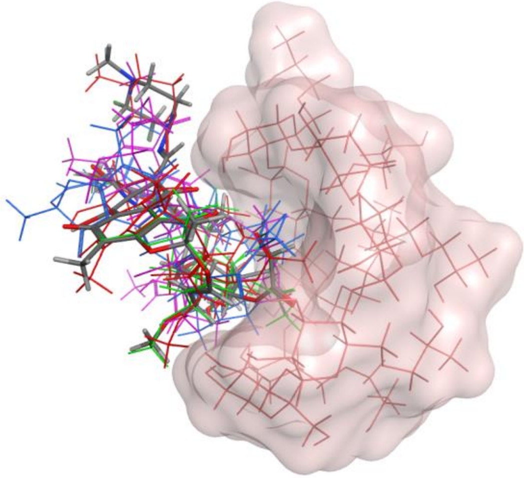

[a]Best pose representative conformation displayed as solid sticks (C= gray; H= white; N= blue; O= red). [b] Explicit solvent approach. [c] closed conformation.
